# Therapeutic Management of Rare Primary Ovarian Neoplasms: Carcinosarcoma, Leiomyosarcoma, Melanoma and Carcinoid

**DOI:** 10.3390/ijerph18157819

**Published:** 2021-07-23

**Authors:** Mateusz Kozłowski, Katarzyna Nowak, Agnieszka Kordek, Aneta Cymbaluk-Płoska

**Affiliations:** 1 Department of Gynecological Surgery and Gynecological Oncology of Adults and Adolescents, Pomeranian Medical University, Al. Powstańców Wlkp. 72, 70-111 Szczecin, Poland; kn13222@gmail.com (K.N.); aneta.cymbaluk@gmail.com (A.C.-P.); 2 Department of Neonatal Diseases, Pomeranian Medical University, 70-111 Szczecin, Poland; agkordek@pum.edu.pl

**Keywords:** carcinosarcoma of the ovary, leiomyosarcoma of the ovary, ovarian malignant melanoma, carcinoid of the ovary, cancer care, surgery, adjuvant treatment

## Abstract

Carcinosarcoma, leiomyosarcoma, melanoma and carcinoid as primary tumors in the ovary are extremely rare. In this paper, the authors reviewed the literature from 2010 to 2021, based on specific criteria, to analyze the treatment of these rare ovarian neoplasms. We also aimed to verify whether modern therapies have been found in recent years. For this article, 80 papers were finally selected. The vast majority of the articles were clinical case reports. Despite single mentions of new potential pharmacological treatments, surgery (radical or fertility-sparing) is definitely the mainstay of treatment. There are currently no treatment guidelines for these tumors. A review of the literature has revealed the use of various adjuvant treatments. We, therefore, believe that a more detailed understanding of the biology of these tumors is necessary in order to find new target points for treatment. We would like to emphasize the importance of creating an international database of rare ovarian tumors which would make it possible to gather data from various oncological centers and enable further research into these neoplasms.

## 1. Introduction

The most common malignancy of the ovary is high grade serous carcinoma. Due to its relatively high prevalence and low cure rate, ovarian cancer is a target for research, allowing the introduction of new drugs or increasing the number of indications of currently used drugs. For a long time, platinum-based chemotherapy was the mainstay of adjuvant treatment. However, as can be observed over the past several years, bevacizumab and poly(ADP-ribose) polymerase (PARP) inhibitors have been included in the treatment of selected groups of patients. Such progress in treatment is unfortunately not observed in rare primary ovarian neoplasms.

Primary carcinosarcoma (PCSO), leiomyosarcoma (PLSO), melanoma (POM) and carcinoid (PCO) of the ovary, although each rare, together form a group of much more common neoplasms. These malignancies occur mainly in peri- and postmenopausal women. Symptoms are non-specific and include among others abdominal pain, distension and weight loss. PCSO, PLSO and POM are often diagnosed at an advanced stage, sometimes with the presence of metastases. Carcinoid, on the other hand, is usually limited to the adnexa and shows a more benign course. The final diagnosis is made on the basis of histopathological examination of the tumor. However, it should be emphasized that the diagnosis is usually confirmed by immunohistochemical examination, which, with a vague histological pattern, is often conclusive. There are no treatment guidelines for these tumors. In the absence of protocols, treatment is often decided individually, in some cases based on the treatment regimen for ovarian cancer. Although in carcinoid treatment, surgery is sufficient in most cases, in three other neoplasms adjuvant treatment is also often used.

The purpose of the review was to analyze the treatment of rare primary ovarian neoplasms. We included carcinosarcoma, leiomyosarcoma, melanoma and carcinoid. In particular, we wanted to analyze whether treatment had changed in recent years and whether there were new treatments for these tumors, so we sought articles from 2010 to May 2021. We conducted a literature search on PubMed, Google Scholar and Cochrane Library. We searched article titles for phrases such as: ‘ovarian melanoma’, ‘leiomyosarcoma of the ovary’, ‘leiomyosarcoma of ovary’, ‘carcinosarcoma of the ovary’, ‘carcinosarcoma of ovary’, ‘carcinoid of the ovary’, ‘carcinoid of ovary’. We have classified articles describing primary tumors, focusing on the treatment and allowing the tracing of the treatment course. We excluded review articles, posters, articles, for which the full text could not be obtained, studies that are not published in English, duplicate articles.

The last search was developed on 18 May 2021. Finally, 80 articles were selected for review.

## 2. Primary Carcinosarcoma of the Ovary (PCSO)

### 2.1. Characteristics

Carcinosarcoma of the ovary is a very rare neoplasm accounting for 1–4% of cancers of this organ [1,2,3]. It is also known as malignant mixed Müllerian tumor (MMMT) or malignant mixed mesodermal tumor [3,4,5]. This type of sarcoma usually occurs in postmenopausal women with the history of low parity [4,6,7,8,9]. The average age of occurrence is 65 years [10]. We have analyzed 14 cases of carcinosarcoma. A total of nine patients were postmenopausal and five of them were premenopausal. The mean age of the tumor incidence in the analyzed group was 56.6 years. Parity was given in 8 articles and 5 (62.5%) patients turned out to be nulliparas. Primary ovarian carcinosarcoma is very aggressive and usually diagnosed at Stage III or IV (75–80%) according to International Federation of Gynecology and Obstetrics (FIGO) [3,8,10,11]. In approximately 90% of carcinosarcoma cases, dissemination beyond the ovary is present at diagnosis [3,8,10]. Carcinosarcoma initially spreads intra abdominally mainly to omentum, upper and lower gastrointestinal tract, bladder, liver and spleen. It rarely gives distant metastases to supraclavicular lymph nodes, bone, lung or the brain [7]. This neoplasm has very poor prognosis with overall survival time 8–32 months [11,12]. The five-year survival depending on the stage is as follows: Stage I 65.2%, Stage II 34.6%, Stage III 18.2%, and Stage IV 11.2% [2].

Tumors are composed of epithelial and sarcomatous elements. The epithelial part is usually composed of serous, endometrioid, or undifferentiated adenocarcinoma. It can also be squamous cell carcinoma or clear cell adenocarcinoma [1,7,12,13]. Sarcomatous elements may contain mesenchymal tissue normally present in the ovary–homologous tissue such as endometrial stromal sarcoma, fibrosarcoma, and leiomyosarcoma or not native to the ovary–heterologous such as chondrosarcoma, rhabdomyosarcoma, lipoma, osteosarcoma or angiosarcoma [1,4,7,10,11,12,13,14]. There are several theories of the pathogenesis of carcinosarcoma. One of them called the combination theory says that both components of a cancer originate from a common epithelial stem cell that undergoes divergent differentiation. Collision theory states that components of carcinosarcoma are of different origin. The other theory, the conversion theory, states that the sarcomatous component develops from the carcinoma during the evolution of the tumor [4,15].

Patients present with symptoms similar to those of epithelial ovarian cancer. These are very non-specific symptoms such as abdominal pain, distension, nausea, vomiting, and weight loss [1,4]. In the analyzed group of patients the most common symptoms were abdominal pain and distension. They also had symptoms such as loss of weight (in three patients), difficulty in breathing (in three patients), one patient complained of constipation and another of weakness and fatigue. One patient developed abnormal vaginal bleeding. The above symptoms are non-specific and may not suggest ovarian cancer. This may be the reason that ovarian cancers are diagnosed at Stage III or IV. Because of overlapping clinical and radiological features, it is problematic to diagnose carcinosarcoma and to differentiate it from epithelial tumors. Additionally, in patients with carcinosarcoma tumor marker CA 125 (cancer antigen 125) usually increases but may also remain normal. Besides, it is not characteristic of carcinosarcoma only and it may increase in other clinical situations. Among the 14 patients analyzed, CA 125 levels were tested in 13 patients. In 12 it was elevated. The lowest was 79.9 U/mL and the highest was 4872 U/mL. In one patient, the CA 125 level was normal–22.2 U/mL. In summary, the diagnosis of carcinosarcoma should be approached comprehensively. Symptoms, radiological findings and tumor marker levels should be considered. However, the final diagnosis can be made only after the histopathological examination of the material taken during the surgery. Immunohistochemistry may also be used in the diagnostics [3,4,10]. Due to the diversity in histopathological structure of the described neoplasm, no characteristic immunohistochemical pattern was found among the analyzed cases. Nevertheless, some cases were found positive for: epithelial membrane antigen (EMA), cytokeratin and vimentin.

Usually, ovarian carcinosarcomas are not involved in inherited cancer syndromes. Carnevali et al. described two cases of carcinosarcoma that occurred in women with hereditary cancer syndromes. One case in a woman with a mutation in the BRCA1 gene, the other in a woman with Lynch syndrome. These two cases show that if carcinosarcoma occurs in young age and there is a family history of multiple cancers a hereditary cancer susceptibility syndrome may be suspected [15].

D’Amati et al., described the first case of mesonephric-like carcinosarcoma in the ovary. It was previously described in vagina, cervix or endometrium. Mesonephric-like carcinosarcoma originate from the remnants of the Wolff ducts and contain a malignant mesenchymal component. Their pathogenesis is uncertain. The authors describe the case of a 74-year-old female patient with abdominal discomfort in whom a tumor in the left ovary was found. The patient underwent exploratory laparotomy to collect material for histopathological examination. After diagnosis, the woman received neoadjuvant chemotherapy (carboplatin and paclitaxel). Then she underwent hysterectomy with left salpingo-oophorectomy, large-bowel segmental resection, omentectomy and resection of two peritoneal nodules. It was the first described case of this rare carcinosarcoma in the ovary. Additional studies containing a larger number of cases must be conducted to determine the origin of this cancer and guidelines for its treatment [16].

### 2.2. Treatment

Because carcinosarcoma is such a rare tumor, no clear guidelines have yet emerged for how to treat it [4,5,17]. Cytoreductive surgery followed by paclitaxel and/or platinum-based chemotherapy is considered the optimal and most effective treatment for ovarian MMMT [3,13]. It has been shown that maximal cytoreduction in patients with advanced stage carcinoma has benefits and improves prognosis and total cytoreduction should be the aim of every surgery [1,10,11,18]. Optimal cytoreduction is considered to be an important determinant of survival [2,11,12]. Systemic chemotherapy is used as adjuvant treatment for carcinosarcoma. Due to the small number of cases of this cancer, it is difficult to determine what chemotherapeutic regimen should be used to treat carcinosarcoma. Therapeutic regimens can be simplified into platinum-based and those not containing platinum [1]. An overall response rate of 68% was reported in patients treated with platinum-based regimens compared with a response rate of 23% in patients treated with non-platinum regimens [19]. The usual treatment is platinum-based chemotherapy with taxane. However, given the effectiveness of ifosfamide in the treatment of uterine carcinosarcoma, some recommend its use in the chemotherapy of ovarian carcinosarcoma [2].

In all analyzed cases surgery was performed. The mainstay of treatment in most patients (11 out of 14) was hysterectomy and bilateral salpingo-oophorectomy. One patient had only bilateral salpingo-oophorectomy, one had unspecified management of the uterus and adnexa and one of them had unilateral salpingo-oophorectomy. These surgeries were extended with removal of all macroscopically visible lesions–cytoreduction. Eight patients underwent omentectomy and one patient underwent omentum biopsy, 5 patients underwent peritoneal biopsies. A total of 3 patients underwent appendectomy. In addition, adjoining gut was removed in two patients and one patient underwent partial resection of small intestine and low anterior resection of the rectosigmoid colon. Additionally, pelvic and/or para-aortic lymphadenectomy was performed in several patients. Adjuvant chemotherapy was given to 10 patients. A total of two of them received platinum-based chemotherapy, six of them were given platinum and paclitaxel. One patient was treated with the combination of platin, paclitaxel and ifosfamide and one patient was treated with platin, etoposide and peplomycin sulfate. Eight patients treated with adjuvant chemotherapy were alive at the time of writing.

One patient in the surgical-only treatment group received neoadjuvant chemotherapy with paclitaxel and platinum. Chemotherapy was given because of lung metastases and was intended to relieve the patient’s breathing problems. Platin and paclitaxel were also administered before the surgery in one patient who was subsequently treated with surgery and adjuvant chemotherapy.

One patient who underwent surgery alone had a recurrence after 6 months. Recurrence was treated with chemotherapy with platinum and paclitaxel. The patient then underwent surgery and repeated chemotherapy using the same regimen. The woman was alive at the time of writing and her overall survival was 72 months. One recurrence also occurred in the group of patients treated surgically with subsequent chemotherapy. She was treated initially with platinum and paclitaxel chemotherapy followed by surgery. After surgery, the same chemotherapy was administered. The relapse occurred 29 months after diagnosis. Overall survival of this patient was 46 months. Results from the Phase III NRG Oncology clinical trial comparing paclitaxel plus carboplatin to paclitaxel plus ifosfamide in women with Stages I–IV recurrent carcinosarcoma of the uterus or ovary show, among other things, that treatment with paclitaxel plus carboplatin is associated with longer progression-free survival than treatment with paxlitaxel plus ifosfamide [20].

Due to the small size of the analyzed groups of patients it is difficult to determine whether surgery alone or surgery followed by chemotherapy have a better survival (Table 1).

Among the reviewed articles, Patnayak et al., in a 2015 article mention the search for new treatments for carcinosarcoma. They describe the potential use of humanized anti-Trop-2 antibody in patients with refractory carcinosarcomas overexpressing Trop-2. They also mention that Human epidermal growth factor-2/neu may be a new target for immunotherapy [3]. However, the methods described are not mentioned in any of the other articles analyzed in this review.

## 3. Primary Leiomyosarcoma of the Ovary (PLSO)

### 3.1. Characteristics

Primary ovarian sarcomas account for less than 3% of primary ovarian malignancies [21]. Of these, leiomyosarcoma accounts for less than 0.1% [21,22,23,24]. In this review, we have analyzed 10 cases of patients diagnosed with leiomyosarcoma. The incidence of ovarian sarcoma to ovarian carcinoma is estimated to be 1:40 [21,23,25]. Leiomyosarcoma most often occurs in postmenopausal women [22,25,26,27,28] with a mean age of 52.6 years [29], however, analysis of selected cases shows that it can also affect younger premenopausal women (mean age of leiomyosarcoma onset in the analyzed cases is 49, 6) [22,25,26,27]. These tumors are usually unilateral and grow to a large size [21,22,25]. The symptoms of this sarcoma are not specific and in the analyzed group were mainly abdominal pain, but also abdominal distention, loss of appetite, difficulty in micturition or intermenstrual bleeding. Due to the presence of vague and non-specific symptoms leiomyosarcoma is diagnosed at an advanced stage and often with distant metastases mainly to lungs and liver [21,22]. Recurrences within the first year after diagnosis are common, mainly in the pelvis and abdomen [30].

The histogenesis of this neoplasm remains ambiguous as ovary does not contain smooth muscles and there are many possible locations from which the neoplasm may develop [26]. These include: totipotent ovarian mesenchyme, smooth muscle fibers of ovarian ligaments, the vascular wall, Wolfian duct remnants, smooth muscle metaplasia of ovarian stromal or theca cells or smooth muscle cells that migrate from within the uterus [25,26,29,30]. Leiomyosarcoma can also develop within teratoma, papillary serous cystadenocarcinoma or serous cystadenoma [26]. Depending on its origin, this type of sarcoma can be divided into three types: teratoid, mesenchymal and Müllerian. Lesions of mesenchymal origin are more common in postmenopausal women and are more likely to metastasize, whereas those of teratoid origin occur in younger women and are usually unilateral [23,28]. In assessing the origin of the tumor immunohistochemical profile may be helpful. Immunoexpression of smooth muscle markers between leiomyosarcomas of vascular origin and those of non-vascular origin have been described. It appeared that tumors of vascular origin showed positivity for h-caldesmon and focally positivity or negativity for desmin while those of non-vascular origin were negative for h-caldesmon with variable levels of desmin expression [29]. Immunohistochemistry is also and mainly used in the diagnosis of this type of sarcoma. The main markers used in the diagnostic process of leiomyosarcoma are: desmin, vimentin, smooth muscle actin, S-100, caldesmon [24,27,28]. In some cases—estrogen, progesterone receptors, p-53 and bcl-2 may be positive [27]. Analysis of selected cases allows us to conclude that all patients showed positivity for smooth muscle actin, seven showed positivity for desmin (only seven were examined) and six showed positivity for vimentin (only six were examined). Thus, it can be confirmed that these are immunohistochemical markers important for the diagnosis of leiomyosarcoma. The importance of immunohistochemistry in diagnostics is also indicated by the fact that imaging examinations such as ultrasonography and its impedance value are not accurate in diagnosing ovarian tumors. Under normal conditions, a high impedance value (RI > 0.6) indicates that a mass is rather benign, while a low impedance value (RI < 0.4) indicates that a mass is more likely to be malignant. He et al., noted that this is not so obvious with ovarian tumors. Ultrasound examination of the described patient showed impedance value (RI = 0.77) indicating that lesion is benign while Doppler ultrasound indicated that the lesion was rather malignant [26]. The above case shows that ultrasound is not an ideal diagnostic method for leiomyosarcoma. What is also valid is that there are no serum tumor markers specific for this sarcoma [28]. An important element used in diagnostics is the assessment of mitotic count. A mitotic index of ≥5 mitotic figures per 10 high powered field (HPF) in the presence of significant atypia has been proposed as a guiding principle for a diagnosis of leiomyosarcoma. Among our cases, in all patients in whom mitotic rate was determined its value was higher than 5 mitotic figures per 10 high powered field. Divya et al., indicate that in case of myxoid leiomyosarcoma the mitotic rate should be determined separately in the solid and myxoid areas because in their case mitotic count in myxoid areas was 3–4 per 10 HPF while in solid areas it was 10–12 per HPF [25]. In conclusion, histopathological examination of the lesion, determination of the mitotic index and immunohistochemistry are significant in the diagnostics of ovarian leiomyosarcoma [26]. To assess staging of leiomyosarcoma it is recommended to use the classification system International Federation of Gynecology and Obstetrics (FIGO) as for epithelial ovarian carcinomas [25,28,30]. Prognosis depends on stage, tumor size, mitotic index, grade, capsular invasion but is rather poor and up to 80% of patients with Stages II–IV die within one year after diagnosis [23,25,28,30]. Patients often die of disseminated metastases but also because of extensive local disease and its pressure effect over abdominal organs [23]. Early diagnosis and optimal cytoreductive surgery are crucial for patients and may improve survival [24,30].

### 3.2. Treatment

Because primary leiomyosarcoma of the ovary is such a rare tumor, there are no specific guidelines outlining its treatment [26,27]. Surgical treatment is now the mainstay of management. Both fertility sparing procedures and surgeries consisting of total abdominal hysterectomy, bilateral salpingo-oophorectomy, omentectomy and excision of the tumor masses are performed [22,27,28]. Chemotherapy or radiotherapy are used as adjuvant treatment even though their effectiveness has not been proven [27,28]. Postoperative radiotherapy may be effective in local control of the disease but does not prevent the development of distant metastases [24,30]. There are also no specific chemotherapy regimens used to treat this type of neoplasm. All patients we have analyzed underwent surgical treatment (surgical treatment of one of them was not described). Regardless of the patients’ age, in all cases the decision was made to perform radical surgery rather than fertility-sparing surgery. In five of them adjuvant treatment was completely abandoned. In other cases, postoperative chemotherapy was used or planned to be used. As there are no recommendations for postoperative chemotherapy, there is no established drug regimen. Among the analyzed patients, docetaxel and gemcitabine were used in two of them. In the third patient who received adjuvant treatment, vincristne, epirubicin and cyclophosphamide were used. Another one was treated with doxorubicin, isofosfamide and mesna. Due to the small size of the group, it is difficult to say whether surgical treatment alone or surgery with adjuvant chemotherapy has a longer survival (Table 2).

Two patients developed recurrence. In one, only after surgical treatment, in another after surgical treatment supplemented with chemotherapy. The treatment of recurrence in the first patient included cytoreductive surgery and chemotherapy, so management of recurrence is very similar to that of the primary tumor. The longest recurrence-free survival was in a 27-year-old patient undergoing both surgery and chemotherapy. Her overall survival by the time the paper was written was 47 months. The shortest overall survival was one month and death was due to postoperative complications (the patient developed sepsis with multiorgan failure and died from septic shock) [29].

## 4. Primary Ovarian Melanoma (POM)

### 4.1. Characteristics

Melanoma is an extremely rare malignancy of the ovary. It occurs more frequently in the ovary as a metastatic neoplasm [31] and much less frequently as a primary neoplasm [32]. The first case was described by Andrews in 1901 [33,34,35]. The literature review includes 18 cases since 2010. Melanoma accounts for 3% of the malignant tumors of female reproductive system and is generally seen in the vulva, vagina and cervix and less commonly seen in the uterus body and ovary [34,36]. It is most common in postmenopausal women in the age group between 50 and 60 years [34,37]. The mean age of POM in the group of patients we analyzed was 51.4 years, which corresponds to the previously described cases. However, it should be noted that in 8 of 18 cases, the age of the women was below 50 years, and the youngest patient was 13 years old at the time of diagnosis [33,34,37,38,39,40,41]. Thus, it should be emphasized that even among adolescent girls, special oncological vigilance should be shown.

Symptoms are usually non-specific, arising from the presence of a tumor in the pelvis minor and general malignancy and include general weakness, loss of appetite, weight loss, abdominal distension, early satiety, lower abdominal pain, lower back pain, abdominal fullness, although the course may also be asymptomatic [31,41,42]. The first symptoms may be organ-specific and related to the metastatic focus. In the case of a patient with brain metastases, the first symptoms were headache, dizziness, nausea, vomiting and photophobia [38]. In 10 of the 18 analyzed cases, patients had metastases involving the pelvic organs, bowel, appendix, omentum, brain, lung, liver and adrenal gland already at the beginning of the diagnostic process [33,35,36,38,39,40,41,42].

In most cases, POM coexists with teratoma. It is the neoplastic transformation of melanocytic teratoma cells that is the most likely etiopathogenesis of POM. However, in some cases the development of POM is unclear. This applies to patients in whom no teratoma component is detected on histopathological examination [33,38,41,43]. The ovary does not contain melanocytes, so the development of melanoma in the absence of a teratoma component is controversial. This may be justified by the fact that despite the suspicion of a primary ovarian cancer, it is a metastasis and in these cases, it is crucial to search for a primary localization outside the ovary. If a primary lesion is not detected, there are also hypotheses that assume the presence of extraovarian melanoma in the past and its regression or metastasis from an unknown primary origin [31,33,38]. However, as totipotent cells are present in the ovary [26], the authors of this review have also questioned whether primary ovarian melanoma could develop precisely from these cells, without then developing a teratoma component. Although this theory would require a detailed analysis of the tumor biology.

As we mentioned, POM most often manifests as an ovarian tumor. The initial diagnosis, in addition to a physical gynecological examination, includes transvaginal ultrasound, which, depending on the stage of the disease, reveals a tumor of the adnexa or a mass in the pelvis minor. Usually, the diagnosis is supplemented by a computed tomography (CT) scan of the pelvis minor in order to analyze the structure of the tumor in more detail. The diagnosis of whether there is a primary or secondary tumor involves a thorough examination including the skin, mucous membranes and the choroid membrane of the eye. After excluding the primary lesion outside the ovary, histopathological examination of the material taken intraoperatively is necessary for the final diagnosis. A characteristic feature of most melanoma cells is the possession of melanin intracellularly, but the amelanotic melanoma should also be remembered [40]. In both cases, immunohistochemical assay, which shows immunopositivity for HMB45, S100, vimentin, Melan-A, is crucial in making the diagnosis [36,38,39,40,44]. All analyzed cases had confirmed melanoma with selected immunohistochemical markers.

### 4.2. Treatment

As the case study shows, the treatment of POM has changed a little since 2010. Currently, there are no guidelines for the therapeutic management. This is related to the fact that POM is extremely rare, and it is difficult to assemble a sufficiently large group of patients for clinical trials. Surgery continues to be the mainstay of treatment. We have observed a varying range of operations in female patients. In most cases, bilateral salpingo-oophorectomy and hysterectomy were performed. Extending the surgery with resection of further organs was tailored individually to each case and included omentectomy, pelvic and para-aortic lymphadenectomy, appendectomy, anterior resection of colon, adrenalectomy. In one case left oophorectomy was performed [41]. In three other cases also no hysterectomy was performed, but only bilateral salpingo-oophorectomy in one case, in another case extended by omentectomy. In the third case of a 13-year-old girl lymphadenectomy and omentectomy were also performed [36,38,40]. Preoperative treatment in one patient included intraperitoneal chemotherapy and immunotherapy (cisplatin and interleukin-2) and intravenous chemotherapy (paclitaxel and cisplatin) [36]. Complementary treatment includes intravenous chemotherapy (mostly platinum-based) and immunotherapy (interferon-α). These drugs are used in various combinations [31,33,35,36,41,42,44]. Hormonotherapy like tamoxifen is used much less frequently [41]. Local radiotherapy is also used to treat metastases [35,38]. The variety of adjuvant therapies shows that there is no specific therapeutic regimen. However, it should be noted that there were more recurrences in the group of patients treated with surgery only, than in the group of patients treated with surgery and adjuvant therapy (Table 3).

One patient was treated for recurrence with combination immunotherapy with ipilimumab (monoclonal antibody to cytotoxic T-lymphocyte associated antigen 4 (CTLA4)) and nivolumab (monoclonal antibody to programmed death 1 (PD-1)). However, disease progression was still observed. The treatment was changed to chemotherapy with dacarbazine, but the patient died 14 months following her initial emergency presentation [39]. Despite treatment of primary cutaneous melanoma involving multiple drug-points, POM still appears to be a malignancy for which there is no effective treatment. It seems important to have a detailed understanding of the biology of this tumor, including differences with malignant melanoma occurring in other locations. However, a novel Insulin-like growth factor II mRNA-binding protein 1 (IMP1) inhibitor–2-{[(5-bromo-2-thienyl)methylene]amino} benzamide (BTYNB)–described in 2017 seems promising. It inhibits cell proliferation and anchorage-independent growth of IMP1-positive melanoma and ovarian cancer cells [45]. We think that in the absence of effective therapy for POM, this treatment could also be considered in patients with this disease.

## 5. Primary Carcinoid of the Ovary (PCO)

### 5.1. Characteristics

Carcinoid of the ovary can occur as a primary or metastatic neoplasm. Primary carcinoid of the ovary is extremely rare. It occurs with a frequency of 0.5% to 1.7% of all carcinoids and about 1% of ovarian cancer [46]. There are 4 main histological types: insulinar, trabecular, strumal, mucinous, but also a variant with several components can occur, i.e., mixed carcinoid [47,48,49,50]. Insular is described as the most common type, but was not observed in the analyzed group [51,52]. PCO is most common in peri- and post-menopausal women [53]. The mean age among the patients was 45.5 years. However, we observed the occurrence of the neoplasm in 14- and 18-year-old girls [49,54].

Carcinoid belongs to the group of neuroendocrine neoplasms (NEN). It develops in most cases from cells in the gut wall. It also occurs, although less frequently, in other organs, including the lungs, mediastinum, thymus, liver, pancreas, bronchi, ovaries, prostate and kidneys. Most tumors grow slowly and have a benign course, with only a small percentage progressing aggressively with the presence of metastases at the time of diagnosis [55]. In the group of 41 cases analyzed, only 5 patients had metastases at the time of diagnosis [56,57,58,59,60].

Carcinoid can be asymptomatic [53,61] as well as with the presentation of symptoms. The cause of symptom presentation may be the presence of a tumor in the pelvis and its effect on surrounding tissues, but also the effect of specific substances produced by tumor cells, or the presence of metastases. Presenting symptoms include abdominal distention, weight loss, abdominal pain or discomfort and loose stools [46,56,62,63]. Note also that persistent, severe constipation may be a symptom. A likely cause of this is the action of peptide YY, which exerts an inhibitory effect on the peristaltic actions of the distal intestine. Interestingly, as described by Noh et al., after surgery, the patient’s constipation resolved rapidly [64]. Carcinoid heart disease is also present in some cases. As shown in the case reported by Buda et al., the patient presented with severe dyspnea and peripheral oedemas. The woman had a large pericardial effusion with compression of cardiac chamber, which required an evacuative pericardiocentesis. In such cases, it is extremely important to avoid a diagnostic delay and to rapidly implement both symptomatic and causal treatment [65]. Clinically, neuroendocrine symptoms such as persistent facial flushing and episodes of hypertension may also be observed [66]. In addition, carcinoid may also present with virilization symptoms [67,68].

The primary diagnosis of carcinoid is mainly based on imaging studies, initially using ultrasound and then MRI (magnetic resonance imaging) and/or CT. The imaging study highlights the tumor of the adnexa. Obtaining tissue material and undergoing histopathological examination is necessary to make a diagnosis. Kumar et al. describe a case in which they performed fine-needle aspiration cytology in the preoperative management to assess cytomorphological features. However, also in this case, surgery was necessary to make a definitive diagnosis [56]. In the analyzed group, although the histological type was not clearly defined in all cases, the most common type was strumal. It should be noted that in some cases it was a mixed tumor with other histological components of carcinoid. Strumal carcinoid is characterized by the co-occurrence of both carcinoid cells and thyroid tissue in the tumor. Hence, it is referred to as a specific type of mature teratoma. Indeed, 16 patients had a mature teratoma component in their tumor [47,48,49,51,53,63,65,66,69,70,71,72,73]. It should be mentioned that there have been cases of strumal carcinoid without the presence of the teratoma, as well as the teratoma component present in histological types other than strumal. This may indicate one of the theories of the development of primary ovarian carcinoid, according to which the carcinoid would develop from the tissues of the teratoma. To confirm the diagnosis immunohistochemical examination is used, which in the presence of neuroendocrine tumor cells shows positive staining for synaptophysin, chromogranin A, peptide YY, CD56 and in the presence of thyroid tissue also for thyroglobulin and thyroid transcription factor-1 (TTF-1) [74,75,76,77,78].

### 5.2. Treatment

Most carcinoid tumors are diagnosed at an early clinical stage. The mainstay of therapy is surgery, which has not changed over the 11 years we have been analyzing. Of the 41 cases, surgery was the treatment of choice in 40. The essence of surgery is to remove the tumor, which is located in the adnexa. In one patient, only cystectomy was performed without adjuvant treatment and the follow up without recurrence was 24 months [73]. Besides, it should be noted that both uterine-sparing surgeries and those with hysterectomy are used in the treatment. In 19 of the analyzed patients, surgery was performed based on unilateral/bilateral salpingo-oophorectomy (including the cystectomy patient described above), without excision of the uterus. In the remaining 21 cases, hysterectomy was also performed (including one patient due to cervical dysplasia). In both groups, the procedure was extended with resections of other organs, depending on the macroscopic status during abdominal exploration, and included omentectomy, hepatic resection, cholecystectomy, appendectomy, removal of the pelvic and paraaortic lymph nodes. In one patient with liver metastases at diagnosis, after a diagnostic laparotomy, the initial management included cisplatin and etoposide with partial tumor regression, followed by Octreotide LAR. However, 5 years later hepatic and pelvic disease progression occurred. The patient underwent right ovariectomy and peritoneal node dissection. After histological examination, which confirmed a NEN involving the right ovary, with a proliferation index of 10% (Ki-67), she received peptide receptor radionuclide therapy (PRRT) with 177Lu-DOTATATE ([177Lu-DOTA-Tyr3]-octreotate) associated with intra-muscular monthly Octreotide LAR. The follow-up of the patient in good clinical condition was 96 months [59]. Adjuvant treatment was implemented in 5 patients, 3 of whom had distant metastases at diagnosis [57,58,60,71,79]. Patients were treated with: a. cisplatin, ifosofamide, mesna, adriamycin; b. paclitaxel, carboplatin, bevacizumab; c. platinum-based chemotherapy; d. paclitaxel, cisplatin; e. everolimus. Despite the lower number of recurrences after surgical and adjuvant treatment compared to surgical treatment only (1 vs. 3), no longer maximum overall survival was observed in the group treated with surgery and adjuvant therapy compared to the group treated with surgery only (7 months vs. 213 months) (Table 4).

This may be related to the mentioned presence of metastases. The shortest follow up was 20 days, after which the patient died due to sepsis [65].

There are currently no treatment guidelines for NEN of the female genital tract. Therefore, many patients are treated according to protocols used for epithelial cancer of the genital tract and not according to recommendations for NEN of e.g., the gastrointestinal system [80]. As this review indicates, most patients are treated with surgery only. Despite the lack of guidelines, the results of surgical treatment are satisfactory. Different adjuvant treatments were used in the analyzed group. It seems that protocols would be particularly helpful in cases of carcinoid stage with distant metastases.

## 6. Conclusions, Perspectives and Clinical Observations of the Authors

The analyzed neoplasms represent a therapeutic challenge for the coming years. Currently, there are no guidelines for their treatment, and an analysis of articles since 2010 shows that treatment has not changed much. Nor have new treatments been developed that would represent a breakthrough in the treatment of these rare tumors.

For carcinosarcoma, the main treatment is surgery with subsequent chemotherapy. Surgical treatment is an important determinant of survival. Chemotherapy mainly uses platinum with paclitaxel, but also ifosfamide. There was only one mention of new treatment options, where the authors mentioned the potential use of humanized anti-Trop-2 antibody in patients with refractory carcinosarcomas overexpressing Trop-2. They also mention that human epidermal growth factor-2/neu may be a new target for immunotherapy. However, this treatment was not used in any of the reviewed articles, so it is difficult to assess its effectiveness.

Radical or fertility-sparing surgical treatment is also the mainstay of treatment for leiomyosarcoma. Adjuvant treatment in the form of chemotherapy or radiotherapy is also used, despite the lack of evidence for their effectiveness. None of the articles reviewed mentioned research into new treatments or possible treatment prospects.

In the melanoma, where in addition to surgery, adjuvant chemotherapy and radiotherapy, immunotherapy is also used in selected cases. In the absence of an effective treatment, a relatively new molecule could be considered, the novel IMP1 inhibitor, BTYNB, which has shown activity against melanoma and ovarian cancer cells. However, it seems that more research is still needed for effects on ovarian melanoma cells.

Among the tumors described, carcinoid has the best prognosis. Surgical treatment alone is characterized by a long overall survival and there are few recurrences. However, protocols for patients with distant metastases are lacking. Various adjuvant treatments have been used, mainly chemotherapy, but also bevacizumab.

**Figure 1:**
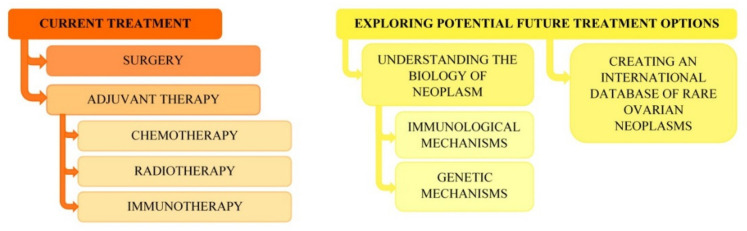
Diagram of current treatment and potential future treatment options in rare primary ovarian neoplasms.

We believe that the biology of rare ovarian cancers, including immunological and genetic mechanisms, should be further studied, which would make it possible to find new drug points. Furthermore, an international database of rare ovarian cancers should be created on the basis of which a thorough analysis could be carried out and, together with a better understanding of tumor biology, new courses of treatment could be identified (Figure 1).

On the basis of this, guidelines could be drawn up, with particular emphasis on individualized treatment.

As this analysis, but also the authors’ own experience, shows, rare cancers occur mainly in peri- and postmenopausal women, but also in younger girls. Cytoreductive surgery is often required, with removal of the adnexa and uterus, which deprives the woman of fertility. This is why we emphasize the importance of appropriate psychological support, both during and after hospitalization. Appropriate patient education and psychological care are very necessary during oncological treatment.

## Figures and Tables

**Table 1:** Comparison of selected features between group treated with surgery only and group treated with surgery and adjuvant chemotherapy in primary carcinosarcoma of the ovary.

Characteristics		Surgical Treatment Only	Surgical Treatment and Adjuvant Chemotherapy
Cases Number (*n* = 14)		4	10
Mean of patient age at diagnosis (min.-max.)		51 years (22–72)	58.8 years (40–80)
Neoadjuvant chemotherapy		1	1
Recurrences number		1	1
Follow up	Death (*n*/min. OS/max. OS)	1/25 mon.	1/46 mon.
	Survival (*n*/min. OS/max. OS)	1/72 mon.	8/6 mon./76 mon.
	Not specified/undergoing treatment (*n*)	2	1

**Table 2:** Comparison of selected features between group treated with surgery only and group treated with surgery and adjuvant chemotherapy in primary leiomyosarcoma of the ovary.

Characteristics		Surgical Treatment Only	Surgical Treatment and Adjuvant Chemotherapy
Cases Number (*n* = 10)		6	4
Mean of patient age at diagnosis (min.-max.)		53.2 years (26–67)	44.3 years (27–65)
Recurrences number		1	1
Follow up	Death (*n*/min. OS/max. OS)	2/1 mon./51 mon.	1/36 mon.
	Survival (*n*/min. OS/max. OS)	1/22 mon.	3/2 mon./47 mon.
	Not specified/undergoing treatment (*n*)	3	0

**Table 3:** Comparison of selected features between group treated with surgery only and group treated with surgery and adjuvant chemotherapy in primary ovarian melanoma.

Characteristics		Surgical Treatment Only	Surgical and Adjuvant Treatment
Cases Number (*n* = 18)		9	9
Mean of patient age at diagnosis (years)		47.6	55.2
Isolated ovarian melanoma		2	2
Associated to ovarian teratoma		7	7
Recurrences number		6	2
Follow up	Death (*n*/min. OS/max. OS)	6/3 mon./17 mon.	5/2 mon./28 mon.
	Survival (*n*/min. OS/max. OS)	1/48 mon.	2/6 mon./12 mon.
	Not specified/undergoing treatment (*n*)	2	2

**Table 4:** Comparison of selected features between group treated with surgery only and group treated with surgery and adjuvant chemotherapy in primary carcinoid of the ovary.

Characteristics		Surgical Treatment Only	Surgical and Adjuvant Treatment
Cases Number (*n* = 40)		35	5
Mean of patient age at diagnosis (min.–max.) (years)		44.3 (14–78)	58.8 (45–85)
Recurrences number		3	1
Follow up	Death (*n*/min. OS/max. OS)	2/20 days/10 mon.	2/24 mon./34 mon.
	Survival (*n*/min. OS/max. OS)	27/2 mon./213 mon.	3/9 weeks/7 mon.
	Not specified/undergoing treatment (*n*)	6	0

## Data Availability

Not applicable.
